# Hydro­flux synthesis and crystal structure of Tl_3_IO

**DOI:** 10.1107/S2056989020012359

**Published:** 2020-09-11

**Authors:** Ralf Albrecht, Heinrich Menning, Thomas Doert, Michael Ruck

**Affiliations:** a Technische Universität Dresden, Chair of Inorganic Chemistry II, Bergstrasse 66, 01069 Dresden, Germany; b Max-Planck Institute for Chemical Physics of Solids, Nöthnitzer Strasse 40, 01187 Dresden, Germany

**Keywords:** crystal structure, hydro­flux synthesis, thallium, oxide iodide, single-crystal XRD

## Abstract

Single-crystals of thallium(I) iodide oxide Tl_3_IO were obtained as by-product in a hydro­flux synthesis at 473 K for 10 h. A potassium hydroxide hydro­flux with a water-base molar ratio of 1.6 and the starting materials TlNO_3_, RhI_3_ and Ba(NO_3_)_2_ was used, resulting in a few black needle-shaped crystals. X-ray diffraction on a single-crystal revealed the hexa­gonal space group *P*6_3_/*mmc* (No. 194) with lattice parameters *a* = 7.1512 (3) Å and *c* = 6.3639 (3) Å. Tl_3_IO crystallizes as hexa­gonal anti-perovskite (anti-BaNiO_3_ type) and is thus structurally related to the alkali-metal halide/auride oxides *M*_3_ *X*O (*M* = K, Rb, Cs; *X* = Cl, Br, I, Au). The oxygen atoms center thallium octa­hedra. The [OTl_6_] octa­hedra share *trans* faces, forming a linear chain along [001]. Twelve thallium atoms surround each iodine atom in an [ITl_12_] anti-cubocta­hedron. Thallium and iodine atoms together form a hexa­gonal close-sphere packing, in which every fourth octa­hedral void is occupied by oxygen.

## Abstract 2

Single-crystals of thallium(I) iodide oxide Tl_3_IO were obtained as by-product in a hydro­flux synthesis at 473 K for 10 h. The oxygen atoms center thallium octa­hedra. The [OTl_6_] octa­hedra share *trans* faces, forming a linear chain along [001]. Twelve thallium atoms surround each iodine atom in an [ITl_12_] anti-cubocta­hedron. Thallium and iodine atoms together form a hexa­gonal close-sphere packing, in which every fourth octa­hedral void is occupied by oxygen.

## Chemical context   

The class of alkali-metal halide/auride oxides comprises several compounds with the general formula *M*_3_ *X*O (*M* = K, Rb, Cs; *X* = Cl, Br, I, Au) (Sitta *et al.*, 1991*a* ▸,*b* ▸; Feldmann & Jansen, 1995*a* ▸,*b* ▸,*c* ▸; Sabrowsky *et al.*, 1996 ▸), Li_3_BrO (Wortmann *et al.*, 1989 ▸), Na_3_ *X*′O (*X′* = Cl, Br) (Sabrowsky *et al.*, 1988 ▸; Hippler *et al.*, 1990 ▸). These ternary oxides crystallize typically as anti-perovskites, *i.e*. in the cubic anti-CaTiO_3_ type. The cesium derivatives Cs_3_BrO, Cs_3_IO and Cs_3_AuO adopt hexa­gonal anti-perovskite structures (anti-BaNiO_3_ type), whereas the Cs_3_ClO crystallizes as anti-NH_4_CdCl_3_ type and thus does not form a perovskite structure. The crystal structure of Rb_3_IO has both face and corner-sharing [ORb_6_] octa­hedra (anti-BaFeO_3–*x*_ type). The adopted structure type depends on the size of the alkali-metal and halide/auride ions and their ratio. The stability range of the different perovskite phases can be estimated by using Goldschmidt’s tolerance factor, where larger *M* and *X* ions tend to destabilize the cubic anti-perovskite structure resulting in the hexa­gonal polymorph (Babel, 1969 ▸; Feldmann & Jansen, 1995*b* ▸).

For the synthesis of the title compound, the hydro­flux method was used, which can be classified as inter­mediate between hydro­thermal and flux synthesis (Chance *et al.*, 2013 ▸). An approximately equimolar mixture of alkali-metal hydroxide (typically NaOH or KOH) and water is used as reaction medium (Albrecht *et al.*, 2020*a* ▸). Good solubility of oxides and hydroxides, highly crystalline reaction products suitable for single-crystal X-ray diffraction analysis, comparably low reaction temperatures and a pressureless setup are essential advantages of the hydro­flux method. In this communication, we report on the synthesis and crystal structure analysis of the thallium(I) iodide oxide Tl_3_IO.

## Structural commentary   

**Figure 1:**
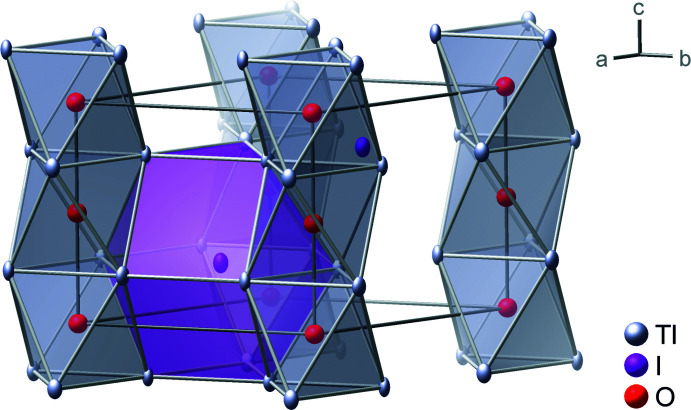
Crystal structure of Tl_3_IO in *P*6_3_/*mmc*, highlighting the one-dimensional chains consisting of [OTl_6_] octa­hedra. Ellipsoids enclose 99% of the probability density of the atoms.

Single-crystal X-ray diffraction on a black needle revealed the composition Tl_3_IO and a hexa­gonal structure in the space group *P*6_3_/*mmc* (no. 194) with lattice parameters *a* = 7.1512 (3) Å and *c* = 6.3639 (3) Å at 100 (1) K. Tl_3_IO crystallizes as hexa­gonal anti-perovskite (anti-BaNiO_3_ type; Fig. 1 ▸, Tables 1 ▸ and 2 ▸). The asymmetric unit consists of three atoms, thallium (site symmetry *mm*2, Wyckoff position 6*h*), iodine (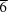 *m*2, 2*d*) and oxygen (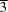 *m*., 2*a*). The oxygen atoms center thallium octa­hedra. The [OTl_6_] octa­hedra share *trans* faces, forming a linear chain along [001]. Twelve thallium atoms surround each iodine atom in a [ITl_12_] anti-cubocta­hedron (triangular orthobicupola). Thallium and iodine atoms together form a hexa­gonal close-sphere packing, in which every fourth octa­hedral void is occupied by oxygen. Thus, also the thallium atom centers an anti-cubocta­hedron, which has the composition [Tl(I_4_Tl_8_)].

The [OTl_6_] octa­hedron is slightly elongated along the chain direction. The O—Tl bond length of 2.549 (1) Å is about 1% longer than in Tl_2_O, at 2.517 (1) Å (Sabrowsky, 1971 ▸). The Tl—O—Tl angles along the chain parallel to *c* are 94.8 (1)°. The shortest Tl⋯Tl distances in Tl_3_IO are with 3.449 (1) Å, very similar to those in thallium metal, which has Tl⋯Tl distances of 3.405 (1) and 3.455 (1) Å in its hexa­gonal sphere packing (Barrett, 1958 ▸). Accordingly, the [ITl_12_] anti­cubocta­hedra are also stretched along [001], with Tl—I distances of 3.576 (1) Å and 3.833 (1) Å. Although thallium(I) has a larger ionic radius (1.70 Å for c.n. = 12; Shannon, 1976 ▸) than potassium (1.64 Å for c.n. = 12), the *M*—O, *M*⋯*M* and the average *M*—I distances in Tl_3_IO are smaller than in K_3_IO by 3.5%, 7.5% and 1%, respectively (Feldmann & Jansen, 1995*b* ▸).

## Synthesis and crystallization   

Thallium(I) iodide oxide, Tl_3_IO, was synthesized in a potassium hydroxide hydro­flux. The reaction was carried out in a PTFE-lined 50 mL Berghof type DAB-2 stainless steel autoclave starting from TlNO_3_ (0.38 mmol; abcr, 99.5%), RhI_3_ (0.06 mmol; abcr, 99%), and Ba(NO_3_)_2_ (0.19 mmol; VEB Laborchemie Apolda, 99%). Water and potassium hydroxide (86%, Fisher Scientific) in the molar ratio of 1.6:1.0 were added to these compounds. The sealed autoclave was heated at a heating rate of 2 K min^−1^ to 473 K and after 10 h cooled to room temperature at a rate of 0.1 K min^−1^. The reaction product after washing with water mainly consisted of thallium(I) iodide, thallium(III) oxide, barium carbonate and a brown powder of an unidentified rhodium-containing compound. A few black single crystals of Tl_3_IO with a needle-like morphology were found, which are sensitive to water and other protic solvents. In contact with water, the Tl_3_IO crystals immediately turn yellow, probably due to the formation of thallium(I) hydroxide and thallium(I) iodide, which are both yellow. Energy-dispersive X-ray spectroscopy on Tl_3_IO single-crystals revealed a disproportionately high oxygen content, indicating surface decomposition.

Several experiments failed to exchange rhodium(III) iodide with other iodine sources like potassium iodide, copper(I) iodide or silver(I) iodide. Likewise, experiments without barium nitrate were not successful. However, when both starting materials were used, Tl_3_IO was obtained reproducibly, also at reaction temperatures of 423 K or 523 K. Similarly, the hydro­thermal synthesis of Na_3_[Tl(OH)_6_] starting from thallium(I) sulfate required heavy metal salts like bis­muth nitrate (Giesselbach, 2002 ▸). The oxidation of thallium(I) to thallium(III) in this reaction was achieved by oxygen in alkaline solutions (Rich, 2007 ▸).

The alkali-metal oxide halides *M*_3_ *X*O are reported to be very sensitive to traces of moisture or carbon dioxide due to their highly basic nature (Feldmann & Jansen, 1995*b* ▸). Remarkably, Tl_3_IO crystallizes in the presence of water from the hydro­flux. In other experiments, we synthesized a water sensitive oxo­hydroxoferrate (Albrecht *et al.*, 2019 ▸) or an oxidation sensitive manganate(V) from hydro­flux (Albrecht *et al.*, 2020*b* ▸). Obviously, the activity of water is dramatically reduced in these aqueous salt melts.

## Refinement   

Crystal data, data collection and structure refinement details are summarized in Table 3 ▸.

## Supplementary Material

Crystal structure: contains datablock(s) I. DOI: 10.1107/S2056989020012359/pk2648sup1.cif

Structure factors: contains datablock(s) I. DOI: 10.1107/S2056989020012359/pk2648Isup2.hkl

CCDC reference: 2030857

Additional supporting information:  crystallographic information; 3D view; checkCIF report

## Figures and Tables

**Table 1:** Atomic coordinates and equivalent isotropic displacement parameters (in 10 ^4^ Å^2^) in Tl_3_IO at 100 (1) K

Atom	Wyckoff symbol	*x*	*y*	*z*	*U*_iso_/*U*_eq_
Tl	6*h*	0.1608 (1)	0.3216 (1)	1/4	47 (1)
I	2*d*	2/3	1/3	1/4	53 (1)
O	2*a*	0	0	0	68 (7)

**Table 2:** Anisotropic displacement parameters (in 10 ^4^ Å^2^) in Tl_3_IO at 100 (1) K

Atom	*U*_11_	*U*_22_	*U*_33_	*U*_23_	*U*_12_	*U*_13_
Tl	38 (1)	24 (1)	73 (1)	0	0	12 (1)
I	42 (1)	42 (1)	77 (2)	0	0	21 (1)
O	68 (10)	68 (10)	68 (19)	0	0	34 (5)

**Table 3:** Experimental details

Crystal data	
Chemical formula	Tl_3_IO
*M*_r_	756.01
Crystal system, space group	Hexagonal, *P*6_3_/*m* *m* *c*
Temperature (K)	100
*a*, *c* (Å)	7.1512 (3), 6.3639 (3)
*V* (Å^3^)	281.85 (3)
*Z*	2
Radiation type	Mo *K*α
μ (mm^−1^)	90.87
Crystal size (mm)	0.09 × 0.05 × 0.03

Data collection	
Diffractometer	Bruker APEXII CCD
Absorption correction	Multi-scan (*SADABS*; Bruker, 2016 ▸)
*T*_min_, *T*_max_	0.113, 0.749
No. of measured, independent and observed [*I* > 2σ(*I*)] reflections	15853, 480, 442
*R*_int_	0.049
(sin θ/λ)_max_ (Å^−1^)	0.995

Refinement	
*R*[*F* ^2^ > 2σ(*F* ^2^)], *wR*(*F* ^2^), *S*	0.018, 0.041, 1.29
No. of reflections	480
No. of parameters	10
Δρ_max_, Δρ_min_ (e Å^−3^)	2.80, −2.01

Computer programs: *APEX3* and *SAINT* (Bruker, 2016 ▸), *SHELXT* (Sheldrick, 2015*a* ▸), *SHELXL* (Sheldrick, 2015*b* ▸) and *OLEX2* (Dolomanov *et al.*, 2009 ▸).

## supplementary crystallographic information

### Crystal data

**Table:**

Tl_3_IO	*D*_x_ = 8.908 Mg m^−^^3^
*M**_r_* = 756.01	Mo *K*α radiation, λ = 0.71073 Å
Hexagonal, *P*6_3_/*m**m**c*	Cell parameters from 8223 reflections
*a* = 7.1512 (3) Å	θ = 3.2–46.5°
*c* = 6.3639 (3) Å	µ = 90.87 mm^−^^1^
*V* = 281.85 (3) Å^3^	*T* = 100 K
*Z* = 2	Needle, black
*F*(000) = 608	0.09 × 0.05 × 0.03 mm

### Data collection

**Table:**

Bruker APEXII CCD diffractometer	442 reflections with *I* > 2σ(*I*)
φ and ω scans	*R*_int_ = 0.049
Absorption correction: multi-scan (SADABS; Bruker, 2016)	θ_max_ = 45.0°, θ_min_ = 3.3°
*T*_min_ = 0.113, *T*_max_ = 0.749	*h* = −14→14
15853 measured reflections	*k* = −11→14
480 independent reflections	*l* = −11→12

### Refinement

**Table:**

Refinement on *F*^2^	0 restraints
Least-squares matrix: full	*w* = 1/[σ^2^(*F*_o_^2^) + (0.0193*P*)^2^] where *P* = (*F*_o_^2^ + 2*F*_c_^2^)/3
*R*[*F*^2^ > 2σ(*F*^2^)] = 0.018	(Δ/σ)_max_ < 0.001
*wR*(*F*^2^) = 0.041	Δρ_max_ = 2.80 e Å^−^^3^
*S* = 1.29	Δρ_min_ = −2.01 e Å^−^^3^
480 reflections	Extinction correction: SHELXL-2016/6 (Sheldrick 2015b), Fc^*^=kFc[1+0.001xFc^2^λ^3^/sin(2θ)]^-1/4^
10 parameters	Extinction coefficient: 0.0035 (2)

### Special details

**Table:**

Geometry. All esds (except the esd in the dihedral angle between two l.s. planes) are estimated using the full covariance matrix. The cell esds are taken into account individually in the estimation of esds in distances, angles and torsion angles; correlations between esds in cell parameters are only used when they are defined by crystal symmetry. An approximate (isotropic) treatment of cell esds is used for estimating esds involving l.s. planes.

### Fractional atomic coordinates and isotropic or equivalent isotropic displacement parameters (Å^2^)

**Table:**

	*x*	*y*	*z*	*U*_iso_*/*U*_eq_	
Tl	0.16078 (2)	0.32156 (2)	0.250000	0.00465 (5)	
I	0.666667	0.333333	0.250000	0.00534 (7)	
O	0.000000	0.000000	0.000000	0.0068 (7)	

### Atomic displacement parameters (Å^2^)

**Table:**

	*U*^11^	*U*^22^	*U*^33^	*U*^12^	*U*^13^	*U*^23^
Tl	0.00378 (6)	0.00238 (6)	0.00733 (9)	0.00119 (3)	0.000	0.000
I	0.00418 (9)	0.00418 (9)	0.00765 (18)	0.00209 (5)	0.000	0.000
O	0.0068 (10)	0.0068 (10)	0.0068 (19)	0.0034 (5)	0.000	0.000

### Geometric parameters (Å, º)

**Table:**

Tl—Tl^i^	3.4494 (3)	Tl—Tl^vi^	3.7538 (1)
Tl—Tl^ii^	3.7538 (1)	Tl—Tl^vii^	3.7018 (3)
Tl—Tl^iii^	3.7538 (1)	Tl—Tl^viii^	3.7018 (3)
Tl—Tl^iv^	3.4494 (3)	Tl—O	2.5490 (1)
Tl—Tl^v^	3.7538 (1)	Tl—O^ix^	2.5490 (1)
			
Tl^i^—Tl—Tl^iv^	60.0	O—Tl—Tl^viii^	132.580 (2)
Tl^iv^—Tl—Tl^iii^	62.648 (2)	O^ix^—Tl—Tl^viii^	132.580 (2)
Tl^vii^—Tl—Tl^vi^	90.0	O^ix^—Tl—Tl^iv^	47.420 (2)
Tl^i^—Tl—Tl^viii^	180.0	O^ix^—Tl—Tl^i^	47.420 (2)
Tl^v^—Tl—Tl^iii^	115.917 (5)	O—Tl—Tl^iii^	42.580 (2)
Tl^iv^—Tl—Tl^viii^	120.0	O—Tl—Tl^iv^	47.420 (2)
Tl^ii^—Tl—Tl^v^	149.235 (3)	O^ix^—Tl—Tl^vii^	132.580 (2)
Tl^i^—Tl—Tl^vii^	120.0	O—Tl—Tl^vii^	132.580 (2)
Tl^iii^—Tl—Tl^vi^	149.235 (3)	O—Tl—Tl^i^	47.420 (2)
Tl^iv^—Tl—Tl^vii^	180.0	O—Tl—Tl^v^	108.774 (4)
Tl^iv^—Tl—Tl^vi^	90.0	O—Tl—Tl^ii^	42.580 (2)
Tl^viii^—Tl—Tl^vii^	60.0	O—Tl—Tl^vi^	108.774 (4)
Tl^v^—Tl—Tl^vi^	54.704 (4)	O^ix^—Tl—Tl^vi^	42.580 (2)
Tl^i^—Tl—Tl^ii^	62.648 (2)	O^ix^—Tl—Tl^iii^	108.774 (4)
Tl^i^—Tl—Tl^iii^	90.0	O—Tl—O^ix^	77.242 (5)
Tl^iv^—Tl—Tl^ii^	90.0	Tl^x^—O—Tl^i^	94.839 (4)
Tl^viii^—Tl—Tl^iii^	90.0	Tl^x^—O—Tl^iii^	85.161 (4)
Tl^viii^—Tl—Tl^ii^	117.352 (2)	Tl—O—Tl^x^	180.0
Tl^ii^—Tl—Tl^iii^	54.704 (4)	Tl^iv^—O—Tl^i^	85.161 (4)
Tl^vii^—Tl—Tl^ii^	90.0	Tl—O—Tl^ii^	94.840 (4)
Tl^i^—Tl—Tl^vi^	62.648 (2)	Tl^iv^—O—Tl^iii^	94.839 (4)
Tl^i^—Tl—Tl^v^	90.0	Tl^x^—O—Tl^ii^	85.160 (4)
Tl^viii^—Tl—Tl^vi^	117.352 (2)	Tl^ii^—O—Tl^i^	94.839 (4)
Tl^iv^—Tl—Tl^v^	62.648 (2)	Tl—O—Tl^iv^	85.160 (4)
Tl^ii^—Tl—Tl^vi^	115.917 (5)	Tl—O—Tl^iii^	94.840 (4)
Tl^viii^—Tl—Tl^v^	90.0	Tl^x^—O—Tl^iv^	94.840 (4)
Tl^vii^—Tl—Tl^v^	117.352 (2)	Tl^ii^—O—Tl^iii^	85.161 (4)
Tl^vii^—Tl—Tl^iii^	117.352 (2)	Tl^ii^—O—Tl^iv^	180.0
O^ix^—Tl—Tl^ii^	108.774 (4)	Tl^i^—O—Tl^iii^	180.0
O^ix^—Tl—Tl^v^	42.580 (2)	Tl—O—Tl^i^	85.160 (4)

Symmetry codes: (i) −*y*, *x*−*y*, *z*; (ii) *x*−*y*, *x*, −*z*; (iii) *y*, −*x*+*y*, −*z*; (iv) −*x*+*y*, −*x*, *z*; (v) *y*, −*x*+*y*, −*z*+1; (vi) *x*−*y*, *x*, −*z*+1; (vii) −*x*+*y*, −*x*+1, *z*; (viii) −*y*+1, *x*−*y*+1, *z*; (ix) −*x*, −*y*, *z*+1/2; (x) −*x*, −*y*, −*z*.
